# P039 Temporal artery biopsy–confirmed giant cell arteritis presenting with ocular ischaemic changes and subsequent progression from smouldering myeloma to multiple myeloma

**DOI:** 10.1093/rap/rkag113.069

**Published:** 2026-11-03

**Authors:** Hiba Salih, Asma Qazi, Ei Ei Phyu Htut

**Affiliations:** Rheumatology, Cambridge University Hospital, Cambridge, United Kingdom; Rheumatology, Cambridge University Hospital, Cambridge, United Kingdom; Rheumatology, Cambridge University Hospital, Cambridge, United Kingdom

## Abstract

**Introduction:**

Giant cell arteritis (GCA) is the most common large-vessel vasculitis in adults over 50 years of age and is associated with significant morbidity, including irreversible visual loss. Increasing evidence suggests an association between GCA and haematological malignancies, including plasma-cell dyscrasias. Ocular ischaemic manifestations of GCA may occur in the absence of classic arteritic anterior ischaemic optic neuropathy (AION), creating diagnostic challenges, particularly when concurrent paraproteinaemia is present.

**Case description:**

A 66-year-old man with no significant past medical history presented with a four-week history of acute-onset occipital headache, scalp tenderness, left-sided jaw claudication, and marked fatigue. He denied visual symptoms. Examination demonstrated scalp tenderness, with intact temporal artery pulses and no focal neurological deficits. Laboratory investigations revealed markedly elevated inflammatory markers, macrocytic anaemia, and thrombocytosis.

Ophthalmological assessment demonstrated retinal nerve fibre layer swelling in the left eye, consistent with a cotton wool spot, without optic disc oedema, in keeping with GCA-associated retinopathy without AION. He was treated urgently with intravenous methylprednisolone followed by oral prednisolone, with rapid clinical and biochemical improvement.

During diagnostic work-up, serum protein electrophoresis identified a new IgA paraprotein, and serum free light chain analysis demonstrated markedly elevated kappa light chains with a kappa:lambda ratio >110. Although paraproteinaemia raised the possibility of hyperviscosity-related ocular ischaemia, the clinical presentation and steroid responsiveness supported a diagnosis of GCA. Temporal artery biopsy demonstrated intimal thickening with patchy chronic inflammatory infiltrate, confirming GCA.

Bone marrow examination revealed a plasma-cell dyscrasia consistent with smouldering IgA multiple myeloma, without features of hypercalcaemia, renal impairment, anaemia, or bone disease (CRAB). He was managed with haematological surveillance while GCA was treated with corticosteroids and later methotrexate. Serial haematological monitoring demonstrated relatively stable paraprotein levels, with free light chain ratios fluctuating in parallel with corticosteroid tapering.

Over the course of follow-up, whole-body MRI demonstrated progression to active multiple myeloma based on International Myeloma Working Group SLiM criteria, and systemic chemotherapy was initiated. His GCA remained in remission, with no recurrence of ocular ischaemic events.

**Discussion:**

The coexistence of GCA and plasma-cell dyscrasias is uncommon but increasingly recognised. Population-based studies consistently demonstrate a higher incidence of haematological malignancies in patients with GCA compared with matched controls. In a large nationwide cohort study, haematological malignancies occurred in approximately 1.9% of patients with GCA compared with 1.2% of controls, corresponding to an approximate two-fold increased risk, with multiple myeloma occurring in approximately 0.4–0.5% of patients with GCA compared with around 0.2% of controls.

Several mechanisms may underlie this association. Chronic immune activation and dysregulation appear central to both GCA and plasma-cell disorders, with persistent cytokine-driven inflammatory environments potentially promoting clonal plasma-cell expansion and disease progression. These shared pathways provide biological plausibility without implying direct causality.

Ocular involvement represents a particularly challenging area of overlap. Retinal ischaemic changes may occur in GCA without classic AION, reflecting partial or early ischaemia. Similarly, plasma-cell dyscrasias may cause ocular ischaemia through paraprotein-mediated microvascular dysfunction or hyperviscosity. In this case, ocular findings in the absence of AION, combined with significant paraproteinaemia, introduced diagnostic uncertainty regarding the underlying mechanism of ischaemia.

Systemic amyloidosis associated with plasma-cell dyscrasias can also masquerade as vasculitis, emphasising the importance of histological confirmation when atypical features are present. Ultimately, progression from smouldering to active multiple myeloma was guided by established International Myeloma Working Group diagnostic criteria.

**Key learning points:**

• Coexistence of GCA and plasma-cell dyscrasia should prompt consideration of haematological screening in patients with atypical features.

• Paraproteinaemia may mimic or coexist with vasculitic ocular ischaemia, complicating diagnosis in GCA.

• Corticosteroid responsiveness does not exclude concurrent plasma-cell dyscrasia, highlighting the need for multidisciplinary follow-up.

